# Effectiveness of a multimodal training programme to improve general practitioners’ burnout, job satisfaction and psychological well-being

**DOI:** 10.1186/s12875-019-1036-2

**Published:** 2019-11-12

**Authors:** C. Barcons, B. García, C. Sarri, E. Rodríguez, O. Cunillera, N. Parellada, B. Fernández, C. E. Alvarado, C. Barrio, J. C. Fleta, D. Ruiz, R. Torrubia

**Affiliations:** 1 Hospital Sagrat Cor, Serveis de Salut Mental Martorell, Centre de Salut Mental d’Adults del Berguedà, Plaça dels Països Catalans, núm. 4, 2a planta Berga, 08600 Barcelona, Spain; 2 Department of Psychiatry and Forensic Medicine, Universitat Autònoma de Barcelona (UAB), Bellaterra, 08193 Barcelona, Spain; 3 Psychiatry Medical Residency Training Programme, CASM Benito Menni, C/ Dr Pujadas, 36 Sant Boi de Llobregat, 08830 Barcelona, Spain; 4 CASM Benito Menni, St. Boi de LLobregat, C/ Dr Pujadas, 36 Sant Boi de Llobregat, 08830 Barcelona, Spain; 5 Unitat de Suport a la Recerca Costa de Ponent, Institut Universitari d’Investigació en Atenció Primària Jordi Gol (IDIAP Jordi Gol), C/ Bellaterra, 41 Cornellà de Llobregat, 08940 Barcelona, Spain; 6 Quality, Assessment and Technology, Direcció d’Atenció Primària Costa de Ponent, Institut Català de la Salut, C/ Bellaterra, 41 Cornellà de Llobregat, 08940 Barcelona, Spain; 7 Baix Llobregat Centre Primary Care Medical Center. Direcció d’Atenció Primària Costa de Ponent, Institut Català de la Salut, Carrer de Bellaterra, 41, 1r Cornellà de Llobregat, 08940 Barcelona, Spain; 8 Molí Nou Primary Care Medical Center, Direcció d’Atenció Primària Costa de Ponent, Institut Català de la Salut, Ciutat Cooperativa, s/n, Sant Boi de Llobregat, 08830 Barcelona, Spain; 9 Camps Blancs Primary Care Medical Center, Direcció d’Atenció Primària Costa de Ponent, Institut Català de la Salut, Pl. d’Euskadi s/n, Sant Boi de Llobregat, 08830 Barcelona, Spain; 10 Montclar Primary Care Medical Center, Direcció d’Atenció Primària Costa de Ponent, Institut Català de la Salut, C/ Pi i Margall, 115 Sant Boi de Llobregat, 08830 Barcelona, Spain; 11 Vinyets Primary Care Medical Center. Direcció d’Atenció Primària Costa de Ponent, Institut Català de la Salut, Ronda de Sant Ramón, 187 Sant Boi de Llobregat, 08830 Barcelona, Spain; 12 Psychiatry and Medical Psychology, Unitat de Psicologia Mèdica, Department of Psychiatry and Forensic Medicine, Universitat Autònoma de Barcelona (UAB), Bellaterra, 08193 Barcelona, Spain

**Keywords:** Brief systemic therapy, Burnout, General practitioners, Job satisfaction, Primary care, Psychological well-being

## Abstract

**Background:**

The changes in the models of care for mental disorders towards a community focus and deinstitutionalisation might have risen General practitioners’ (GPs) workload, increasing their mental health concerns and the need for solutions. Pragmatic research into improving GPs’ work-related health and psychological well-being is limited by focusing mainly on stressors and through not providing systematic attention to the development of positive mental health via interventions that develop psychological resources and capacities. The aim of this study was twofold: a) to determine the effectiveness of an intensive multimodal training programme for GPs designed to improve their management of mental-health patients; and b) to ascertain if the program could be also useful to improve the GPs management of their own burnout, job satisfaction and psychological well-being.

**Method:**

Eighteen GPs constituted a control group that underwent the routine clinical *Mental health support programme for primary care*. An experimental group (*N* = 20) additionally received a Multimodal training programme (MTP) with an Integrated Brief Systemic Therapy (IBST) approach. Through questionnaires and a clinical interview, level of burnout, professional satisfaction, psychopathological state and various indicators of the quality of administrative and healthcare management were analysed at baseline and 10 months after the programme.

**Results:**

In relation to government of mental-health patients indicators, on the one hand MTP group showed statistically significant improvements in certain administrative health parameters, but on the other it did not improve opinions and attitudes towards mental illness. Regarding GPs management of their own burnout, job satisfaction and psychological well-being assessments, the MTP presented better scores on global psychopathological state and better evolution of satisfaction at work; psychopharmacology use dropped in both groups; in contrast, the MTP did not improve burnout levels.

**Conclusions:**

Findings of this preliminary study are promising for the MTP (with an IBST approach) practice in primary care. More research evidence is required from larger samples and randomized controlled trials to support both the hypothetical adoption of MTP (with an IBST approach) as a part of a continuing professional-training programme for GPs’ management of mental-health patients and its positive effects on work-related health factors.

## Background

Burnout, job satisfaction and a psychological well-being construct are key work-related health factors that need to be assessed and controlled in any work environment, and hence also in primary-care services. Their correct management could improve the quality of healthcare offered, promoting greater patient satisfaction and better treatment compliance, improving morbidity and mortality, as well as reducing the likelihood of hospitalization [1, 2].

Despite variabilities in defining these constructs, links between burnout, job satisfaction and psychological well-being have been established [3–5]. Recent research shows that GPs have a higher prevalence of mental health problems, such as symptoms of burnout, and are less satisfied with their work-life balance than is the case with the general population [6]. Taking into account the discrepancies in results between the longitudinal and cross-sectional surveys, we observe, first, that given the high levels of burnout and frequency and patient safety incidents within primary care, research on this issue is essential. Nonetheless, burnout prevalence fluctuates over time and countries, observing some studies reporting high rates in their samples [7], whereas others describing lower rates [8]; one survey conducted in 13 European countries found a 12% of GPs scoring *high* for all burnout dimensions [9]. Regrettably, according to longitudinal studies, GPs’ burnout prevalence tends to remain relatively stable [10]. Second, despite the contextual differences between longitudinal surveys of GPs’ job satisfaction, available evidence does not confirm a declining degree over recent years despite the aforementioned high rates of burnout and poor mental wellbeing [11–13]. Third, so far less attention has been given to GPs’ psychological well-being, although certain risk factors in GPs’ mental health are indicated by the bibliography, such as lack of reward by patients [14].

Even given the associations found between these three factors, to date there is no sound theory that connects them. Perhaps the strongest proposal would be the *Job demands control model*, since, according to the greater part of occupational stress and health research, most studies of psychosocial factors as antecedents of impaired well-being and work-life imbalance have been based on this model [15].

Attending to the rise in the number of visits, the increasing complexity of clinical work and the scarcity of resources [16], previous levels of burnout, job satisfaction and psychological well-being might change, with a concomitant need for GPs to be better skilled at coping with this increased burden at less personal cost. In this context, a number of limited controlled psychological interventions have already shown certain benefits associated with the improvement of GP burnout, job satisfaction and—especially—psychological well-being. Mindfulness-based programmes may perhaps obtain a prominent status in such contexts, showing short-term benefits in burnout, stress and anxiety levels [17, 18]. Likewise, cognitive-behavioral-based interventions have also displayed short-term improvements in stress outcomes [19, 20]. Recently, a modified mindfulness-based cognitive therapy course also demonstrated the potential to reduce stress and burnout [21]. Furthermore, in a controlled trial evaluating a simple letter, giving feedback and interpreting psychological scores together with a self-help sheet, contributed to a reduction in psychological distress after 3 months [22]. Related systematic reviews and meta-analyses have recommended implementation of the aforementioned approaches, among others [23, 24], but such reviews also questioned the alleged good outcomes due to a detection of various shortcomings. For example, Murray and colleagues conducted a systematic review of 5392 studies related to interventions aimed at improving GPs’ psychological well-being, and detected distinct shortcomings and risks of bias such as insufficient information on the temporal stability of the improvements sought; use of self-administered questionnaires as primary outcome measures; and long-term follow-up of mental illness not being reported, among others [25]. Given these circumstances, it is difficult to ascertain which elements of these therapies are really of use for improving doctors’ work-related health and psychological well-being. Beyond the debate asking which of the array of therapeutic targets are eligible and what their real impacts are, it would seem to be more effective and appropriate to shift from the deficient approach underpinning stress-response improvement towards a more proactive approach for fostering mental health that empowers and enhances work and personal resources [26].

Considering its multiple and complex nature, it is unlikely that a single approach from a given discipline (such as psychology) could be sufficient to effectively address these work-related factors and the psychological well-being of GPs. In this context, *Collaborative Care Models* (CCMs) offers a framework in which related disciplines can be combined, consequently allowing for an evaluation of their respective interventions. CCMs are team-based, multicomponent interventions that have been shown to be cost-efficient in improving mental and physical outcomes for a range of mental-health conditions across diverse populations and primary-care settings [27, 28]. To our knowledge, CCM methodology has never been tested on the burnout, job satisfaction and psychological well-being of GPs rather than that of patients. One possibility to put this bio-psychosocial programme into practice would be to promote GPs’ patient-management strategies and competences for distinct pathologies, most especially for mental disorders. In fact, GP-patient encounters focussing on mental-health concerns represent a large proportion of GPs’ patient lists and workload. In our context, even though this remain under-detected, 1.4 million primary-care patients sought assistance for some type of mental-health problem in Catalonia in 2016, which represented 24% of total primary-care visits [29]. As a comparison, this exceeds the estimated 12.7% of such visits for Australia in 2014–15 [30], for example. To understand this figure, we need to take into account the fact that primary health care is the access to the mental health system for the vast majority of the population in Europe [31]. These numbers may well increase in the near future given the widespread benefits and acceptance of the importance of primary-care-oriented health systems in terms of greater effectiveness, efficiency and equity [32]. It is therefore not surprising that, in light of the high prevalence and disability of mental disorders, recent calls to action for global mental health have been made [33]. GPs will have a key role in this mental-health assistance.

Taking into account the research considerations indicated above, by means of empowering activities and instructions, GPs could enhance their individual and group-management strategies and the competences applicable to mental-health patients in primary care. We should also note that GPs mainly tend to work alone, and also that passive dissemination of guidelines to improve the recognition and management of mental disorders have generally and to date been found to have minimal positive outcomes [34]. In our routine clinical *Mental-health support programme for primary care*, these abilities were taught in an intermittent and unstructured manner, in accordance with both down-top (primary-care-centre coordinators) and top-down demands (GPs’ comments in internal qualitative surveys). Our team was therefore requested to devise strategies for delivering better integrated mental health with less personal cost. As a result, the aim of this study was binary: a) to determine, for the first time, the effectiveness of an intensive multimodal training programme (MTP) with an Integrated Brief Systemic Therapy (IBST) approach for GPs designed to improve their management of mental-health patients; and b) to ascertain if the program could be also useful to improve the GPs management of their own burnout, job satisfaction and psychological well-being. In order to assess the first objective, quality-of-healthcare-management indicators were analysed along with GPs’ opinions about mental illness; to evaluate the second one, in addition to questionnaires, the use of a clinical interview and psychopharmaceutical indicators were also deemed necessary.

## Method

We conducted a quasi-experimental study with two non-randomised groups. Pre and post-intervention measurements were registered among GPs working in the public-health system between January 2016 and February 2017.

### Inclusion criteria

Subjects had to be GPs working in public primary-care units. These units also had to belong to the assistance sector of our ambulatory mental-health service. GPs needed to be willing to attend at least 80% of the training programme and to fulfil the psychometric measures.

### Participants and recruitment strategy

Our public ambulatory mental-health service provided assistance to all four primary-care units in Sant Boi (Barcelona) throughout the routine clinical *Mental-health support programme for primary care.* All GPs involved were invited to participate. In this programme, in order to strengthen cooperation, a mental-health team regularly visited each primary-care unit to conduct assistance, coordination and training tasks. These primary-care units covered a population of 95,313 inhabitants in 2016; such a territorial representation provides patients from urban and semi-urban areas.

After a presentation on the management of psychiatric patients, GPs from all primary care centres were invited to participate in the study. Those who agreed to participate were given the baseline assessment questionnaires. In addition, an interview with an independent rater (Psychiatry Medical Residency Training programme) was scheduled for each participant. During this interview, besides delivering questionnaires, the *Brief Psychiatric Rating Scale* (BPRS) was applied to assess participants’ psychiatric symptoms. BPRS was not used to allocate participants to intervention groups. The rater was trained by a senior specialist in administering BPRS. All interviews were carried out within a maximum period of 15 days from sign-on. The rater was blind to the study’s objectives.

Subsequently, GPs were allocated to the experimental group (EG) or control group (CG). Allocation was not random; instead, it corresponded to the primary-care unit for which the participant was working for. Each group was composed of GPs from 2 primary care services, respectively.

### Intervention design

The CG underwent our clinical routine programme for primary care, titled *Mental-health support programme for primary care*. It was aimed at treating mild mental disorders in primary-care units from a normalizing and preventive perspective. To accomplish these goals, a team of mental-health specialists visited each primary-care setting to provide frequent and direct assistance. These included a psychiatrist regularly performing weekly clinical and advisory functions; a clinical psychologist also performing clinical and advisory tasks (fortnightly); and a nurse providing monthly advisory and training tasks.

In the EG, within the aforementioned programme framework, we added and carried out a multimodal training programme (MTP). The intervention was structured as a continuing-education course with group psychoeducational activities. It was coordinated by the clinical psychologist guided by the psychotherapy model and the CCMs. Course duration was 9 hours in total and consisted of nine weekly sessions. MTP comprised different clinical group sessions (1 h/session), each conducted by the same professional, in this order:

1. *Clinical Psychology*: a senior clinical psychologist carried out six sessions of integrated brief systemic therapy (IBST), which essentially integrates solution-focused and problem-focused models. Training was conducted according to guidelines [35–37]. In the initial session, participants’ attitudes in the management of dysfunctional cases were discussed (for example, paternalistic behavior; difficulties establishing limitations for patients’ requests, etc.). Here, as at the end of each session, the aim was to reach group consensus regarding the given topic, which could then be useful for participants’ work environment. In the remaining sessions, distinct techniques were taught such as defining complaints in specific behavioural terms; investigating unsuccessful attempted solutions to a problem; using the patients’ position to negate problem-maintaining solutions; negotiating specific and positive goals; discussing patients’ ambivalence and resistance management; identifying exceptions to problem sequences; discussing possible pre-treatment improvement; using scaling questions to encourage series of small steps; giving patients credit for their improvements; verbal and non-verbal language techniques.

Real-patient videos were presented, and practical exercises were carried out during the sessions. GPs were also encouraged to bring their own cases of patients who might be experiencing difficulties.

2. *Psychiatry*: a senior psychiatrist carried out two sessions designed to provide instruction on how to correctly conduct a psychopathological examination, while also detailing the areas that need to be examined and the correct terminology to use. The intention was not for GPs to establish a thorough diagnosis but rather for their examinations to be more productive and more accurate. This makes coordination meetings with mental-health services more constructive and is of use for therapeutic planning.
3. *Social work*: a senior social worker carried out one session reporting on social and community services that might be suitable for distinct patients, but most especially for those suffering mental disorders. These services included non-governmental organization, family-patient associations, social clubs, etc.

Finally, regarding non-psychiatric medical conditions, GPs were encouraged to reach a consensus on *what not to do* as a team (as opposed *what to do*, which is already well-established in clinical guidelines), given its low efficacy or efficiency.

All participants in the study were offered the course free of charge.

At the end of the study, the EG went back to monitor only the *Mental health support programme for primary care*. Nonetheless, within the ordinary coordinating meetings with GPs, mental-health specialists continued referring to the programme’s concepts and procedures, and team agreements were made (for example, if the case was already being treated by our mental-health service, assistance was not duplicated in primary care when the reason for consultation was the same). At all events, no formal mandatory supplementary work was issued during or after the training.

The MTP was therefore an ad-hoc clinically rooted training programme for GPs’ management of mental-health patients, although to some extent its psychological dimension could be applicable to all types of cases. It seeks to improve all weaknesses and defects detected by the *Mental-health support programme for primary care* that we were already carrying out. For example, detection of mental disorders, which are often unobserved and/or unrecognised by GPs; shortage of communication and management skills; scarcity of teams’ collaborative strategies, etc. These shortcomings were factors affecting GP burnout, job satisfaction and well-being, which were the ultimate targets that we intended to address.

### Outcome measures

Outcome measures are shown in Table 1.

**Table 1:** List of Outcome measures

Outcomes	Instruments
Management of mental-health patients indicators	
Administrative and healthcare indicators	(a) *Total annual visits for all pathologies;* (b) *Rate (percentage) of annual visits linked to Mental Health;* (c) *Rate of Accessibility*
Opinions about Mental Illness	*Struening and Cohen’s Opinion about Mental Illness questionnaire* (OMI) (38)
Burnout, job satisfaction and psychological well-being indicators	
Job Satisfaction	*Font-Roja Job Satisfaction Questionnaire* (FR) (39)
Burnout	*Maslach Burnout Inventory* (MBI) (40)
Psychological well-being	*Brief Psychiatric Rating Scale* (BPRS) (41)
Psychopharmacology use	Current use of psychiatric medication; asked within the clinical interview

We administered validated versions of these instruments for both EG and CG to each participant, selecting those that were most used in the related Spanish bibliography in order to facilitate any comparison:

1. Self-reported psychometric measures:
  - Socio-demographic questionnaire designed ad hoc for this study. This included 8 items related to personal, social and work information
  - Psychopharmacology use: Within the clinical interview, GPs were asked about the current use of psychiatric medication.
  - *Struening and Cohen’s Opinion about Mental Illness questionnaire* (OMI) [38]. The Spanish adaptation has shown satisfactory global reliability (*Cronbach’s alpha =* .81)*.* It has 63 items, yielding five standardized opinion-attitude factor scores: *Negativism* (α = .81); *Social/interpersonal* (α *=* .70)*; etiology* (α = .71); *Authoritarianism* (α = .79)*; Restrictiveness* (α = .68*); Prejudice (α = .69).*
  - *Font-Roja Job Satisfaction Questionnaire* (FR) [39]. The *extended version* presents adequate psychometric properties (*Cronbach’s alpha* = .79). It has 26 [1–5] Likert-type items. It contains 9 job satisfaction dimensions: *Satisfaction at work; Work tension; Professional competence; Work pressure; Professional promotion; Interpersonal relationship with superiors; Interpersonal relationships with peers; Extrinsic characteristics of status; Labor monotony.*
  - *Maslach Burnout Inventory* (MBI) [40]. The Spanish version corresponds to the later renamed *MBI-Human Service Survey* (MBI-HSS). It has shown adequate psychometric properties within its three dimensions of burnout: *Emotional exhaustion* (EE) (*Cronbach’s alpha* = .89), *Depersonalization* (DP) (α = .57); and *Personal accomplishment* (PA) (α = .72). *Total MBI score* is obtained as: EE + DP + LPA. LPA (*lack of personal accomplishment*) is calculated as: [LPA = 48 - PA]. The cut-off points for MBI that we used were those proposed by Doulougeri and colleges in 2016: EE scores ≥27 are considered as *high*, 19–26 as *average*, ≤ 18 as *low;* DP scores ≥10 as *high,* 6–9 as *average*, ≤ 5 as *low*; PA scores ≥40 as *high*, 34–39 as *average*, ≤ 33 as *low*; Total MBI scores percentile > 76 as *high*, 25–75 as *average*, < 25 as *low*.
2. Hetero-applied psychometric measures:
  - *Brief Psychiatric Rating Scale* (BPRS) [41]. This semi-structured interview is one of the oldest and most widely used scales by clinicians and researchers to measure psychiatric symptoms. It presents adequate psychometric properties (*Cronbach’s alpha* for *Positive scale* α = .73; *Negative scale* α = .83; *Global psychopathology* α = .87). We used the 18-item version. Single items are rated on a Likert-type scale (1, *not present*; 2, *very mild*; 3, *mild*; 4, *moderate*; 5, *moderately severe*; 6, *severe*; 7, *extremely severe*). The range of possible BPRS total scores is [18–126], where a higher score indicates more psychiatric symptoms.

As regards administrative and healthcare indicators, these were provided by the regional health authority. For the baseline data, these indicators were registered in 2015 and 2016 (and extracted in 2016 and 2017, respectively, for the current study). For the post-intervention data, we used the following as indicators of the objective work burden for each GP: (a) *total annual visits for all pathologies*; (b) *rate (percentage) of annual visits linked to mental health*; (c) *rate of accessibility*: annual percentage of patients’ attempted requests for visits for the following 48 h and that were successfully scheduled for each professional. In all three cases, data on patients enrolled for home-care programmes, chronically complex patients and patients with chronic advanced diseases were rejected.

Follow-up assessments of GP status took place 10 months after finishing the programme. This post-programme assessment timing was average or higher than that observed in other related research [17, 18].

### Sample size

No sample-size calculation was formally determined. Given our GP population (*N* = 45), we attempted to recruit as many participants as possible. From initial estimations with 45 GPs, we were aware that we would be able to detect a 0.856 effect size in a t-test; we therefore only had the power to detect large effect sizes and through a single bivariate contrast. Our team nevertheless deemed this worthwhile in order not to achieve robust data which reinforce strong claims, but rather to serve as a pilot study for evaluating MTP potential.

Considering the observed data as the real latent distribution and rectifying threshold levels of significance from 0.05 to 0.0026 through Bonferroni’s adjustment for multiple comparisons, in *Results* we indicate the power to detect as significant for the intervention effect in the observed pre-post change for the primary outcome measures in linear mixed-effects models (LMMs).

### Data analysis

Sample characteristics were described by calculating medians and inter-quartile ranges (IQR) for numerical variables, and absolute and relative frequencies (%) for binary and categorical variables. Baseline group differences were evaluated using the Wilcoxon Test for numerical variables and Fisher’s exact test for binary and categorical variables.

Paired Wilcoxon Signed Rank Tests were performed to compare repeated intra-group measurements. To check for differences between groups in progression, we fitted unadjusted linear mixed-effects models (LMMs)—as a longitudinal proxy to bivariate analysis—to account for the longitudinal data assessments and the complexity of inter-group interactions, regressing the different outcomes for treatment group, follow-up, and their interaction. The results are shown in terms of *p*-values of the marginal effects, representing the significance of baseline differences between treatment groups, overall change over time in the overall sample, and differences in evolution over time between treatment groups, respectively. In the tables, in addition to the p-value and where this is significant (*P*-value of ≤0.05 was used as the cut-off point), association direction is also shown. For *Inter-group differences*, a *+* sign indicates a higher score or a higher evolution in the EG; in the global change analysis, this sign indicates a higher score in the follow-up assessments.

The main outcome measures included in the analysis were all the administrative and healthcare indicators, as well as the overall job satisfaction, burnout and well-being. They are key work-related health factors that need to be assessed and controlled, according to related surveys.

All statistical analyses were conducted using the R software package (v. 3.2.2; R Development Core Team, 2015).

## Results

### Participants

Thirty-eight out of forty five GPs agreed to participate in the study (four GPs were dismissed because they were the authors of this study and the rest declined to collaborate). Finally, 18 GPS were allocated to CG, and 20 to EG. All 38 GPs completed follow-up measures after two GPs were rejected (one from each group) due to retiring from their jobs during this period.

### Baseline patient-demographic values

Groups were homogeneous in terms of age (*p* = 0.177), years of professional experience (17[12.25, 28]; *p* = 0.349) and sex (*p* = 0.045), where men were slightly more numerous in EG. Likewise, no statistically significant differences were detected between proportions of indefinite contracts (*p* > 0.999), mixed shifts (*p* = 0.291) and hours of mental-health training during the last year (*p* = 0.129), variables that were respectively predominant in both groups. No statistically significant differences between groups were observed for length of time on current contract (*p* = 0.037), this being higher in CG.

### Changes in outcome measures within and between groups

The average attendance at the nine sessions was 75% (70%, clinical psychology; 90%, psychiatry; 65% for the social work). Attendance rates at coordinating meetings or other activities that constitute the *Mental-health support program for primary care* were not registered for either group.

**Fig. 1:**
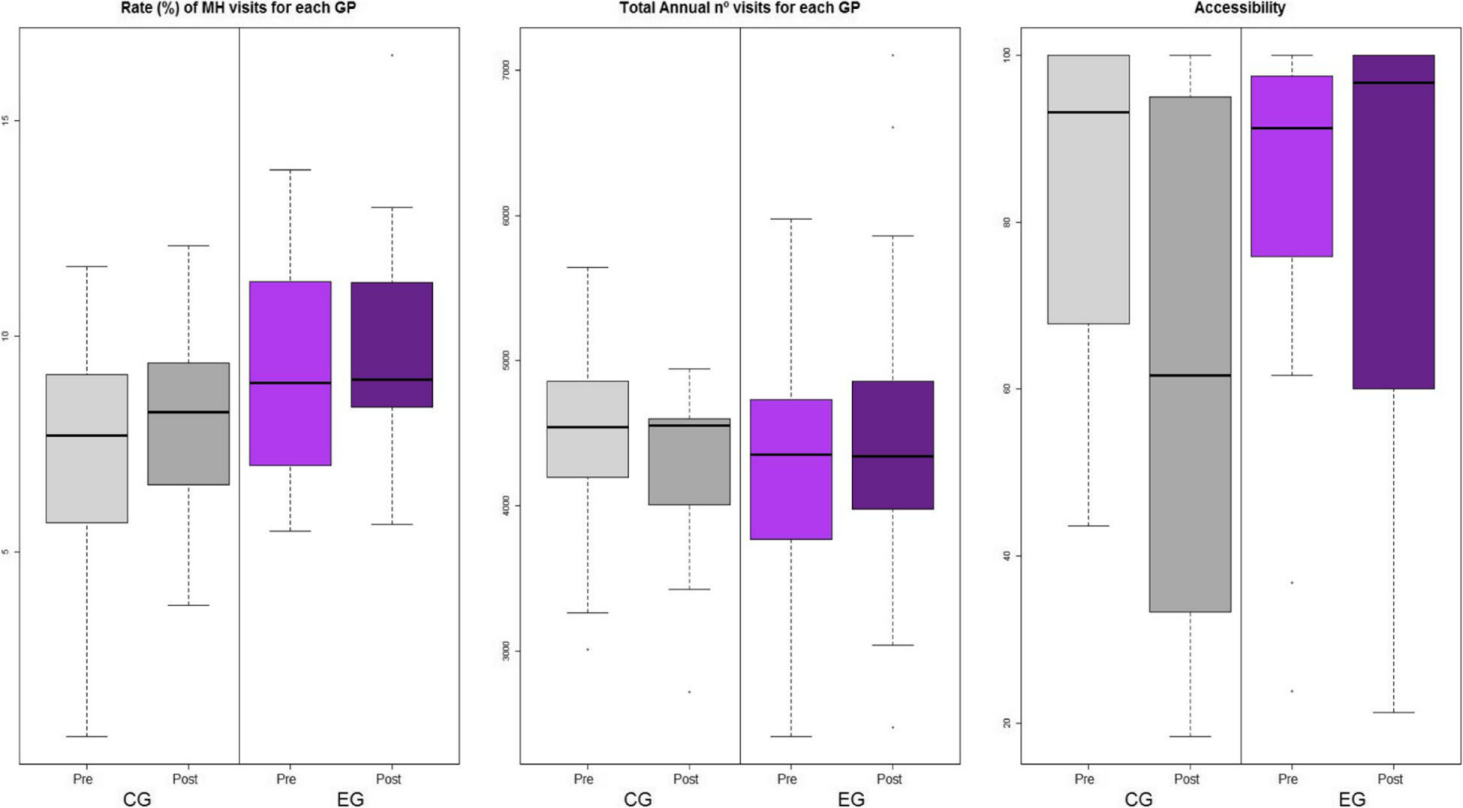
Distribution (box-plots) of administrative parameters’ reported by 38 general practitioners, by intervention group (CG, control group; EG, experimental group), and before and after intervention (Pre, pretreatment; Post, Post-treatment)

In relation to government of mental-health patients indicators, as regards administrative data (Fig. 1), first, no statistically significant differences between groups were detected for the percentage of *annual visits by each GP linked to mental health*, either in *overall pre-post change* (*p* = 0.360) or in inter-group evolution differences (*p* = 0.544). Second, we did observe a lower rate of *total annual visits by each GP for all pathologies* in the CG when examining the *overall pre-post change (p* < 0.003), although this result was not regarded as significant after Bonferroni’s adjustment; besides, it was identified a greater inter-group evolution differences in the EG (*p* < 0.001). Finally, rate of *accessibility* decreased in the CG when observing *overall pre-post change* (*p* < 0.001), which was exactly the opposite to the evolution of the EG, thus contributing to the significance of the inter-group evolution differences in *accessibility* (*p* < 0.001). No significant baseline inter-group differences were detected in any of the previously commented variables, except for *total annual visits by each GP linked to Mental Health*, this being higher in the EG (*p* = 0.040).

As regards opinions and attitudes towards mental illness reported in the *Struening and Cohen’s Opinion about Mental Illness questionnaire* (OMI), a worsening of all five dimensions was observed in *overall pre-post change* for both groups (Table 2), the EG presenting only a different evolution in prejudice, namely a higher increase (*p* = 0.004+).

**Table 2:** Non-adjusted evolution analysis of Struening and Cohen’s Opinion about Mental Illness questionnaire’s punctuations of 38 GPs depending on intervention group (2016–2017)

	Control Group Evolution *N = 18*			Experimental Group Evolution *N = 20*			LMMs *p*-values^a^		
	Pre	Post	*W*	Pre	Post	*W*	Baseline^b^	Change^c^	Evol.^d^
F1	59 [57.25, 60.75]	73 [71.25, 78]	0^e^	58 [53.75, 61.25]	72 [68, 77.50]	0^e^	0.633	<0.001 + ^e^	0.889
F2	44 [40, 49]	53.50 [50.25, 57]	0^e^	42.50 [33.75, 48]	58 [50, 62]	0^e^	0.311	<0.001+ ^e^	0.163
F3	9 [8, 11]	18.50 [17.25, 21.50]	0^e^	10 [7.75, 12.25]	18 [17, 21]	0^e^	0.983	<0.001+ ^e^	0.867
F4	8.50 [6.25, 10]	11.50 [11, 14]	0^e^	7 [6, 9]	11 [10, 13]	0^e^	0.555	<0.001+ ^e^	0.303
F5	7.50 [6, 9]	10.50 [9, 12]	0^e^	6.50 [5, 8]	12 [9.50, 13]	0^e^	0.337	<0.001+ ^e^	0.004

Abbreviations: *Pre* Pretreatment, *Post* Post-treatment, *W* Wilcoxon Signed Rank Tests

Struening and Cohen’s Opinion about Mental Illness questionnaire’s factors: F1: Negativism; F2: Social/interpersonal etiology; F3: Authoritarianism; F4: Restrictiveness; F5: Prejudice

^a^linear mixed-effects models; ^b^Inter-group baseline differences; ^c^Pre-post Overall Change; ^d^Inter-group Evolution differences

^*e*^*p < 0.0026* (intervention group versus control group)

Regarding GPs management of their own burnout, job satisfaction and psychological well-being indicators, with respect to the responses from the *Font-Roja Job-Satisfaction Questionnaire* (FR), no significant differences were detected between groups in terms of overall job satisfaction (Table 3). However, we did observe a decrease in *satisfaction at work* in the CG*,* reflected both in *overall pre-post change* (*p* = 0.009), although again this result was not regarded as significant after Bonferroni’s adjustment; besides, it was identified a greater inter-group evolution differences in the EG (*p =* 0.002). Likewise, a decrease in *interpersonal relationship with peers’* scores was observed in CG (*p* = 0.011), but it did not reach statistical significance.

**Table 3:** Non-adjusted evolution analysis of *Font-Roja Questionnaire*’s punctuations of 38 GPs depending on intervention group (2016–2017)

	Control Group Evolution *N = 18*			Experimental Group Evolution *N = 20*			LMMs *p*-values^a^		
	Pre	Post	*W*	Pre	Post	*W*	Baseline^b^	Change^c^	Evol.^d^
F1	14 [10.50,16]	12 [9, 6]	0.031^e^	15 [13, 16.25]	16 [15, 17]	0.065	0.304	0.009	0.002+ ^e^
F2	13.50 [12.25, 16.75]	15 [1, 16]	0.773	12 [11, 14]	13 [11, 16]	0.418	0.188	0.876	0.587
F3	4.50 [3, 6]	5 [4, 6]	0.404	5 [4, 6]	5 [4.50, 6]	0.689	0.386	0.130	0.512
F4	12 [12, 13]	11 [11, 12]	0.499	12 [10, 12.25]	12 [10,12.50]	0.904	0.543	0.618	0.728
F5	11 [10, 13.75]	12 [11, 15]	0.303	12 [9, 13]	12 [10, 13]	0.631	0.736	0.099	0.336
F6	6 [4, 6.75]	6 [4, 8]	0.510	4 [4, 6.50]	4 [4, 6.50]	0.753	0.156	0.567	0.934
F7	5 [5, 6]	6 [5, 7.]	0.095	6 [5.75, 7]	5 [5, 6]	0.057	0.032	0.122	0.011
F8	5 [5, 6]	6 [4, 6]	0.525	5.50 [4, 6]	5 [5, 6.50]	0.104	0.864	0.502	0.577
F9	5 [4, 5.75]	4 [3, 5]	0.050	4.50 [4, 6]	5 [4, 5]	0.968	0.665	0.069	0.186
Total	76 [73, 80.50]	77 [75, 78]	0.726	75 [72, 77.50]	76 [73, 83]	0.485	0.818	0.796	0.515

Abbreviations*: Pre* Pre-treatment, *Post* Post-treatment, *W* Wilcoxon Signed Rank Tests

*Font-Roja Questionnaire’s* dimensions: F1: satisfaction at work; F2: work tension; F3: Professional competence; F4: work

pressure; F5: professional promotion; F6: interpersonal relationship with superiors; F7: interpersonal relationships with peers; F8: extrinsic characteristics of status; F9: labor monotony

^a^linear mixed-effects models; ^b^Inter-group baseline differences; ^c^Pre-post Overall Change; ^d^Inter-group Evolution differences

^*e*^*p < 0.0026* (intervention group versus control group)

No significant statistical differences were observed in either the *Total burnout* or in any of the three *Maslach Burnout Inventory* (MBI) parameters (Table 4).

**Table 4:** Non-adjusted evolution analysis of *Maslach Burnout Inventory’s* punctuations of 38 GPs depending on intervention group (2016–2017)

	Control Group Evolution *N = 18*			Experimental Group Evolution *N = 20*			LMMs *p*-values^a^		
	Pre	Post	*W*	Pre	Post	*W*	Baseline^b^	Change^c^	Evol.^d^
EE	24.50 [14, 41.25]	25.50 [18, 34.25]	0.756	19.50 [13.75, 23.25]	21.50 [15.50, 28.25]	0.089	0.038	0.881	0.263
DP	7 [5.25, 13.75]	9 [7.25, 11]	0.835	6 [4, 8.50]	7 [3.75, 12.25]	0.317	0.425	0.535	0.807
PA	38 [33.25, 45.25]	38 [32.25, 43.75]	0.297	38 [34, 42.50]	37.50 [29.75, 43]	0.420	0.559	0.410	0.550
Burn	46.50 [24.50, 62.25]	46 [30, 57.25]	0.727	37 [29, 49.25]	40 [33, 52]	0.331	0.243	0.600	0.637

Abbreviations*: Pre* Pretreatment, *Post* Post-treatment, *W* Wilcoxon Signed Rank Tests

*Maslach Burnout Inventory’s* subscales: EE: *Emotional exhaustion*; DP: *Depersonalization*; PA: *Reduced personal accomplishment*

Global Scales: Burn: *Burnout*

^a^linear mixed-effects models; ^b^Inter-group baseline differences; ^c^Pre-post Overall Change; ^d^Inter-group Evolution differences

^*e*^*p < 0.0026* (intervention group versus control group)

**Fig. 2:**
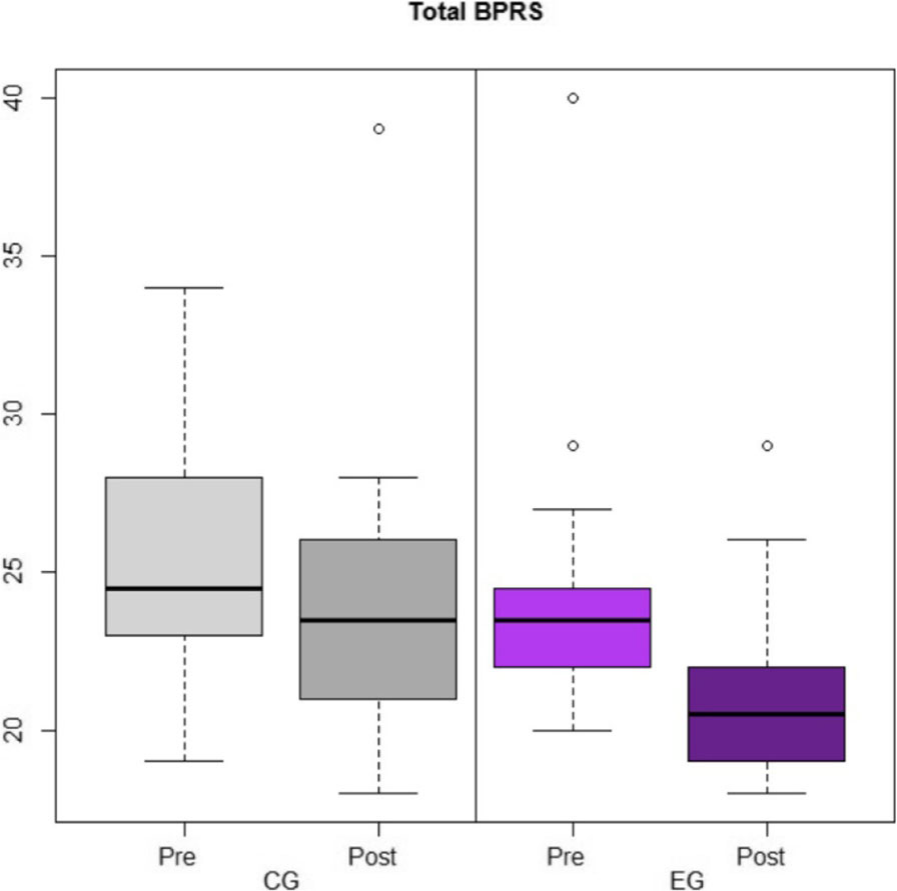
Distribution (box-plots) of *Brief Psychiatric Rating Scale*’s punctuations reported by 38 general practitioners, by intervention group (CG, control group; EG, experimental group), and before and after intervention (Pre, pretreatment; Post, Post-treatment)

As for well-being, we observed statistically significant reductions in *Total Brief Psychiatric Rating Scale* (BPRS) in the EG (baseline: 23.50 [22, 24.25]; post-treatment: 20.50 [19, 22]; *p* = 0.001), while this was not observed in CG (baseline: 24.50 [23.25, 27.75]; post-treatment: 23.50 [21, 26]; *p* = 0.122) (Fig. 2). Nonetheless, in LMMs, no *overall pre-post change (p =* 0.100) or *inter-group evolution differences* (*p =* 0.147) were detected.

Focusing only on both single BPRS item scores or on differences in intervention within the subgroup of *worrying cases* (defined as GPs suffering ≥1 from *moderate* to *extremely severe* symptoms), no statistical differences were observed between groups.

Psychopharmacology use dropped in both groups when observing *overall pre-post change* (*p* = 0.014), falling from 6 to 2 GPs using psychopharmaceuticals in the CG, and from 3 to 2 in the EG. Regarding type of psychopharmaceuticals, this decrease was especially noticeable in the case of benzodiazepine consumption (*p* = 0.037)*.*

Finally, the statistical *power* analysis to detect the intervention effect as significant in the observed pre-post change for the primary outcome measures in LMMs was as follows: *power* = 0.006 for overall job satisfaction; *power* = 0.006 for *Total burnout; power* = 0.013 for *Total Brief Psychiatric Rating Scale.*

## Discussion

Our findings, although insufficient, highlight the potential of MTP (from an IBST approach) to be hypothetically integrated as part of a continuing training programme for GPs’ individual and group-management competences for mental-health patients, which may ultimately enhance GPs’ work-related health and psychological well-being to a certain extent. On the one hand, we observed improvements in the EG for the global psychopathology state, certain administrative parameters and psychopharmacology use, along with better evolution of *satisfaction at work*; on the other hand, MTP did not alleviate *burnout* level or enhance *opinions and attitudes towards mental illness (prejudice)*, which deteriorated in both groups.

### Comparison with existing literature

As with other programmes aimed at engaging GPs in the management of psychiatric patients based on a long-term and sustainable partnership, MTP advances have not so far led to the programme being adopted by the regional Institute of Mental Health [42].

In relation to government of mental-health patients indicators, first, analysing the indicated improvements on our administrative health data offered by the MTP, it is difficult to draw clear conclusions when comparing our results with related surveys, given the disparity of samples, health-system structures, etc. [27]. The achievements shown by the EG in *accessibility* and in *total annual visits by each GP for all pathologies* (inter-group evolution differences) might be interpreted as case-management improvements, although our study design did not allow us to draw strong conclusions or to elucidate alternative explanations. Additionally, the results related to the *rate of annual visits by each GP linked to Mental Health* were inconclusive, which was probably because GPs did not always codify mental-health visits as such.

Two components that could compromise both the work-related health factors and the GPs’ role in diagnosing mental disorders and arranging treatment are (first) the stigmatising attitudes held by GPs towards mental illness, and (second) their reluctance to become involved in shared-care practice [43]. Contrary to other similar studies, MTP did not improve opinions and attitudes towards mental illness [44], although our baseline OMI scores didn’t reflect a high prejudice towards mental disorders but they did show a lack of sufficient dealings in psychopathology which is congruent with previous studies [45]. Although it was not a direct target of our programme, we did expect positive changes as a desirable side effect. Given both the negative evolution of all OMI scores registered in both groups and the positive outcomes of EG on other work-related health parameters, this leads us to question whether opinions and attitudes towards mental illness are a somewhat independent factor. In other words, feeling more capable to cope with mental- health patients may be distinct from our personal thoughts about and interests in this subject. Given our results, more in-depth, longer-lasting and distinct goal-oriented interventions might be needed to reverse these negative attitudes. Following this line of argument, key elements here are the addressing of both level of work pressure and of the low level of training and awareness in relation to mental disorders [46].

Regarding GPs management of their own burnout, job satisfaction and psychological well-being indicators, first, in contrast to other surveys, the MTP did not show better results in overall job satisfaction, nor did it achieve a reduction in GPs’ intention to change their location [20]. We were unable to carry out further analyses with closely related research aimed at enhancing GPs’ mental-health case management within a CCMs because such research failed to assess this issue [42]. Although not globally, EG did present improvements in some of the *Font-Roja* dimensions, reaching statistical significance in *satisfaction at work* (inter-group evolution differences). Clearly, effective inter-professional management of individual patients depends on confidence in one’s colleagues’ skills and good communication, which are issues that are also treated by the MTP. Broader and far more complex interventions are needed in order to address job satisfaction, which in turn is regarded as a key element for resolving the GP recruitment crisis [47].

Contrary to other interventions [17], MTP did not improve burnout levels in our study. Nonetheless, at baseline the median level of burnout (total MBI scores) was 38 (IQR = 29, 54), being this moderate level observed according with previous research [48, 49]; only 2 (5.26%) GPs reported a total MBI scores percentile > 75 (as *high*), figure less prevalent than in other studies [9]. In any case, we consider tackling GPs’ burnout an important issue that should be addressed in future interventions because GPs have high rates of burnout and poor mental wellbeing compared with the general population and other healthcare professionals [49, 50]. Besides, amongst other negative effects, GPs perceive that burnout and poor wellbeing negatively impacts their ability to deliver safe care [51]. We posit that additional resources should be assigned to this subject in order to mitigate our result.

In line with related studies, our results reflected a high proportion of GPs presenting psychiatric symptoms [52]; taking into account only the cases where mental health support could be somewhat recommended, at baseline 21 (55.26%) GPs suffered from *moderate* to *extremely severe* symptoms. Regarding the type of psychiatric symptoms observed, as signaled by the literature, anxiety, depression and somatic concerns were predominant [53]. The MTP improved GPs’ global psychological well-being, but contrary to other studies [17], no *inter-group change* or *evolution differences* were detected. Possible interference may have been given rise to by psychological state being evaluated by a psychiatric interview rather than being self-administered. Furthermore, although score differences for single BPRS items were not statistically significant, a tendency towards greater reductions was detected in the EG.

Finally, so far, we have not found related psychological interventions with which to compare our results in psychopharmacology use. This dropped in both groups, but our study’s limitations again impeded us from making any strong claims with regard to this positive outcome.

### Study strengths and limitations

As limitations of our study, first there is the modest sample size that influences statistical significance and *power*, since statistically significant differences are more difficult to identify in smaller samples. Nonetheless, the study does comprise an important proportion of the total amount of GPs in our health sector. Second, although the administration of our clinical interview (BPRS) could be somewhat controversial in non-clinical samples, it is not limited to indicating only the participants’ own perceptions, as is the case with questionnaires. Response bias (for instance the social-desirability bias) is a widely discussed phenomenon in behavioural and healthcare research where self-reported data are used [54]. Regarding administrative data, although relatively good results were reported for the EG, it is difficult to compare our data externally with other health centres, due to disparity in terms of organization and characteristics. Additionally, indicators themselves are not exempt from criticism. Finally, treatment integrity and fidelity were not assessed or supervised.

As strengths of our study, we would like to emphasise, first, that it was conducted in real clinical practice with all its accompanying constraints: high service demand, work overload, restrictions on the frequency of follow-up meetings, etc. Thus, the duration of our MTP was far from the 50 h or more invested in some other programmes [55]. Second, we evaluated the sustainability of improvements (over nearly 10 months) rather than immediate post-intervention effects, which differs from other studies [17, 22]. Third, GPs were encouraged to introduce suggested MTP procedures into ordinary clinical practice, but in order to avoid selection bias toward only highly motivated professionals no complementary work commitments were set within the inclusion criteria, unlike other studies [17].

### Implication for practice

Finally, it is clear that GP burnout, job satisfaction and psychological well-being require broader and more in-depth approaches than that offered by single disciplines (such as psychology) if we are to improve their institutional and work conditions.

Although further research with methodological improvements and prolonged instruction periods is required, our preliminary findings suggest that this new clinical-rooted MTP (from a BST approach) could have the potential to be adopted as part of a continuing professional-training programme for GPs’ management of mental-health patients. To some extent, it could ultimately enhance GPs’ work-related health factors such as burnout, job satisfaction and psychological well-being. Nonetheless, so far, any strong claim can be stated.

## Conclusions

This study provides inside into GPs’ work-related health and psychological well-being in a period where these aspects might have been worsening. It highlights the need to adopt multimodal training interventions aimed at developing GP’s psychological resources and capacities. This research may help the instructors to recognize the utility of a new intensive training programme for GPs. This personalised approach may assist GPs especially when treating mental-health patients in areas such as on how to correctly conduct a psychopathological examination; on learning verbal and non-verbal language techniques aimed at producing behavioural changes; or on searching for social and community services that might be suitable for distinct patients.

## Data Availability

The datasets generated and/or analysed during the current study are not publicly available due to the *Data* Protection and *Confidentiality Policy*; however, they are available from the corresponding author on reasonable request.

## Abbreviations

BPRS: Brief Psychiatric Rating Scale
BST: Brief Systemic Therapy
CCMs: Collaborative Care Models
CG: Control Group
EG: Experimental Group
FR: *Font-Roja* Job Satisfaction Questionnaire
GP: General Practitioner
MBI: Maslach Burnout Inventory
MTP: Multimodal Training Programme
OMI: Struening and Cohen’s Opinion about Mental Illness Questionnaire
